# Genome Sequence of *Clostridium* sp. Strain P21, a CO-Fermenting Acetogen Isolated from Old Hay

**DOI:** 10.1128/MRA.00864-20

**Published:** 2021-03-18

**Authors:** D. Annie Doyle, Kathleen E. Duncan, Ralph S. Tanner

**Affiliations:** a Department of Microbiology and Plant Biology, University of Oklahoma, Norman, Oklahoma, USA; University of Southern California

## Abstract

Here, we report the genome sequence of *Clostridium* sp. strain P21, isolated from old hay from Stillwater, Oklahoma. This announcement describes the generation and annotation of the 5.6-Mb genomic sequence of strain P21, which will aid in studies targeting genes involved in the enhancement of acid-alcohol production.

## ANNOUNCEMENT

*Clostridium* sp. strain P21 (DSM 111390) is a Gram-positive, strictly anaerobic rod-shaped bacterium isolated from old hay from Stillwater, Oklahoma. Strain P21 was isolated in parallel with Clostridium carboxidivorans P7^T^ (DSM 15243) and “*Clostridium ragsdalei*” P11 (DSM 15248) and enriched with CO at 38°C (1). Axenicity was confirmed via PCR amplification of the regions V1 to V9 of the 16S rRNA gene, as described by Weisburg et al. (2). Strain P21 was selected for whole-genome sequencing to confirm the presence of genes for the Wood-Ljungdahl pathway and acid-alcohol production.

The 16S rRNA gene sequence placed P21 within group 1 *Clostridium* with 97.9% similarity to *C. carboxidivorans* P7^T^, isolated from a settling lagoon (1). Growth characteristics of P21 on CO, on fructose, and on H_2_ are in Table 1. Its growth was similar to that of related acetogens (1). Strain P21 was grown anaerobically in 10 ml of basal medium containing fructose, as described by Liou et al. (1) and centrifuged for 20 min at 6,000 × *g*. Cells were washed, and RLA lysis buffer (Maxwell 16; Promega) was added. DNA extraction was performed using the automated Maxwell 16 tissue LEV total RNA purification kit (Promega) purification system v4.90 modified from the manufacturer’s instructions as described by Oldham et al. (3). DNA library preparation and genome sequencing were performed at Oklahoma Memorial Research Foundation Next-Generation Sequencing Core using the NEBNext Ultra II library prep kit (New England BioLabs) and MiSeq reagent kit v3 (Illumina) on an Illumina MiSeq instrument as per the manufacturer’s instructions. Through the Department of Energy Systems Biology Knowledgebase (KBase) data platform (4), paired-end sequencing reads were trimmed using Trimmomatic v0.36 (5) with TruSeq3-PE, TruSeq2-PE, and NexteraPE-PE adapter files. Further adapters were removed, and low-complexity reads were filtered using Cutadapt v1.18 (6) and PRINSEQ v0.20.4 (7). Quality-filtered paired-end reads were assembled using SPAdes v3.13.0 (8). Contigs with coverage of <120× were removed using Geneious Prime v2020.1.1. Default parameters were used except where noted otherwise. The draft genome consists of 67 scaffolds from 7,670,130 reads (read length, 2 × 300 bp) with a depth of coverage of 405× and an *N*_50_ value of 223,637 bp. Gene prediction and annotation was performed using RAST v0.1.1 (9, 10) and NCBI Prokaryotic Genome Annotation Pipeline (11). The assembled draft genome is 5,645,749 bp with a G+C content of 29.45% and contains 109 RNA genes, including 21 rRNA genes (8 5S, 6 16S, and 7 23S), 80 tRNA genes, and 8 noncoding RNA (ncRNA) genes.

**TABLE 1:** Growth of strain P21 on CO^*a*^

Substrate	Doubling time (h)	Final *A*_600_	Acetate produced (mM)	Butyrate produced (mM)	Final pH
None^*b*^		0.05	3.7	0	6.0^*c*^
Fructose	5.4	0.95	32.6	3.2	4.6
CO/CO_2_	7.8	0.79	28.1	6.5	4.4
H_2_/CO_2_	21.0	0.30	23.8	1.4	4.3

a Methods in Liou et al. (1).

b Base medium contained 1 g/liter yeast extract (1).

c Initial pH 6.1 (1).

Genomic analysis using UniProt (12) and BLAST (13) confirmed the presence of genes in P21 that encode the Wood-Ljungdahl pathway, various acid-alcohol-producing pathways, and an Rnf-complex. We hope that this genome sequence proves useful for finding more information on the genomic potential of this acetogen for acid and alcohol production, particularly for industrial applications.

### Data availability.

The 16S rRNA gene sequence, whole-genome sequence (WGS), and raw sequencing reads for strain P21 were deposited in GenBank and the Sequence Read Archive (SRA) under the accession numbers MT176110, JABBNI000000000, and SRR11451959. The WGS and SRA reports can be found with BioProject number PRJNA613251.
